# Common mycorrhizal networks improve survival and mediate facilitative plant interactions among *Andropogon gerardii* seedlings under drought stress

**DOI:** 10.1007/s00572-025-01181-z

**Published:** 2025-02-03

**Authors:** Margaret Russell, Veronika Řezáčová, Kirby Shane Miller, Wynter Helene Nardi, Morgan Brown, Joanna Weremijewicz

**Affiliations:** 1 Department of Biology, North Central College, 30 N Brainard St., Naperville, IL 60540-5461 U.S.A.; 2 Czech Agrifood Research Center, Drnovská 507, Prague 6, Czech Republic

**Keywords:** *Andropogon gerardii* (Big Bluestem), Arbuscular mycorrhizal fungi, Common mycorrhizal networks, Drought, Phosphorus, Plant survival

## Abstract

Under drought conditions, arbuscular mycorrhizal (AM) fungi may improve plant performance by facilitating the movement of water through extensive hyphal networks. When these networks interconnect neighboring plants in common mycorrhizal networks (CMNs), CMNs are likely to partition water among many individuals. The consequences of CMN-mediated water movement for plant interactions, however, are largely unknown. We set out to examine CMN-mediated interactions among *Andropogon gerardii* seedlings in a target-plant pot experiment, with watering (watered or long-term drought) and CMN status (intact or severed) as treatments. Intact CMNs improved the survival of seedlings under drought stress and mediated positive, facilitative plant interactions in both watering treatments. Watering increased mycorrhizal colonization rates and improved P uptake, particularly for large individuals. Under drought conditions, improved access to water most likely benefited neighboring plants interacting across CMNs. CMNs appear to have provided the most limiting resource within each treatment, whether P, water, or both, thereby improving survival and growth. Neighbors near large, photosynthate-fixing target plants likely benefited from their establishment of extensive hyphal networks that could access water and dissolved P within soil micropores. In plant communities, CMNs may be vital during drought, which is expected to increase in frequency, intensity, and length with climate change.

**Supplementary Information:**

The online version contains supplementary material available at 10.1007/s00572-025-01181-z.

## Introduction

It is projected that climate change will prolong periods of drought and alter precipitation patterns across the globe (Caretta et al. 2022), and although plants can respond to drought stress through morphological, physiological, and biochemical mechanisms (Fang and Xiong 2015), 70% of species additionally associate with arbuscular mycorrhizal (AM) fungi (Brundrett and Tedersoo 2018) that improve water relations during drought (Augé 2001; Augé et al. 2015). In some instances, the presence of AM fungi is detrimental to plant growth (as reviewed by Augé 2001), but much of what we understand about AM functioning during drought has been revealed by studies in which hosts are grown in the presence or absence of AM fungi. Although such studies have been invaluable, we know very little about how interconnecting networks of extraradical AM fungus hyphae, called common mycorrhizal networks (CMNs), influence plant interactions for water (Püschel et al. 2020). It has been established, though, that CMNs have the power to influence plant nutrition and growth, interactions, populations, and communities (Leake et al. 2004; Selosse et al. 2006; Horton 2015; van der Heijden et al. 2015).

The power of CMNs in plant communities lies in their ability to be conduits of belowground biological markets through the partitioning of mineral nutrients among interconnected hosts. According to the largely accepted “reciprocal rewards” hypothesis (Kiers et al. 2011), large C provisioners receive more mineral nutrients, like P or N, in return from AM CMNs compared to small plants (*i.e.* Lekberg et al. 2010; Hammer et al. 2011; Merrild et al. 2013; Zheng et al. 2015; Weremijewicz et al. 2016). CMN-mediated reciprocal rewards amplify competitive interactions among interconnected individuals because large individuals come to dominate a particular resource provided by CMNs, and if the resource is growth limiting, then this exchange suppresses the growth of smaller individuals. Isotopic tracing studies with prairie grasses (*e.g.* Weremijewicz et al. 2016), agricultural (*e.g*. Merrild et al. 2013), and model species (*e.g.* Fellbaum et al. 2014), have indeed demonstrated this intensified, asymmetric, competition by CMNs among interconnected individuals. When large C provisioners are prevented from fixing, and ultimately provisioning C, then little to no ^15^N is obtained from CMNs by hosts (Fellbaum et al. 2014; Weremijewicz et al. 2016).

Occasionally, however, the reciprocal rewards hypothesis is not supported, and other biological phenomena may be at play, such as source-sink dynamics, host-fungus complementarity, and functional differences among different species of AM fungi (Walder and van der Heijden 2015). For example, Walder et al. (2012) found that the largest C provisioner received the least P and N from a CMN comprising a single AM fungus species, and in a follow up study, found that this could be attributed to the regulation of inorganic P transporters with different P affinities when associating with individual species of AM fungi (Walder et al. 2016). CMNs have additionally been found to mediate facilitative interactions. In an arid environment, movement of N from nurse plants to adult plants was detected, although it only made up 2.6% of the total N requirement of individuals (Montesinos-Navarro et al. 2016). CMN-mediated facilitative interactions have also been found among deep and shallow rooted plants, in which deeply rooted individuals hydraulically lift water that is then redistributed by extraradical mycorrhizal fungus hyphae within the soil matrix (Querejeta et al. 2003) and to interconnected shallow-rooted individuals (Egerton-Warburton et al. 2007; Singh et al. 2019).

Much more is known about the role of CMNs in nutrient partitioning than water partitioning. We do know, however, that the presence of AM fungi can improve water uptake for plants through many different indirect mechanisms (Augé 2001). AM fungi improve mineral nutrient uptake for host plants, which ultimately improves physiological aspects of their growth that increase drought resistance, such as an increased expression of aquaporins (Bárzana et al. 2014), stomal conductance (Augé et al. 2015; Symanczik et al. 2018), leaf hydration (Yang et al. 2014) and mineral nutrient uptake (Ouledali et al. 2018). The AM symbiosis also improves plant osmoregulation, which entails actively decreasing plant water potentials to create a gradient that promotes turgor, stomatal opening, and photosynthesis (Ruiz-Lozano 2003; Wu et al. 2017; El-Samad and El-Hakeem 2019). Additionally, associating with AM fungi protects against drought-induced oxidative damage (Ruiz-Lozano 2003; Duc et al. 2018; Zou et al. 2021; Tereucán et al. 2022). AM fungi also alter root morphology by increasing lateral root growth (Gutjahr and Paszkowski 2013) which may benefit plants during drought by increasing the volume of soil for water uptake. Finally, soils with mycorrhizal plants are able to retain water soil moisture better than those with nonmycorrhizal plants, most likely because of the effects on soil structure, such as increased water stable aggregates and extraradical hyphal densities (Augé et al. 2001). The simple physical presence of AM fungus hyphae in the soil increases the hydraulic conductivity of soil (Bitterlich et al. 2018), particularly of loams with low water contents (Pauwels et al. 2023). Soils with AM fungus hyphae have greater water retention capacities than those without AM fungi, most likely because of an increase in pore space heterogeneity (Pauwels et al. 2020). Hyphae also can decrease the resistance of water movement from high to low water potentials and close large gaps among soil pores by growing across them (Bitterlich et al. 2018). Water sticking to the surface of AM hyphae moves through capillary action to host plants (Augé 2004).

Arbuscular mycorrhizal fungus hyphae can also move water through direct mechanisms related to cytoplasmic streaming within hyphae (Allen 2007; Egerton-Warburton et al. 2007; Ruth et al. 2011; Püschel et al. 2020; Pauwels et al. 2020; Kakouridis et al. 2022), but the magnitude of this effect on plants is debated. Püschel et al. (2020) found that although *Rhizophagus irregularis* hyphae doubled the water acquired by *Medicago truncatula*, this was most likely due to more extensive root systems in mycorrhizal plants compared to nonmycorrhizal plants. The direct hyphal acquisition of water was low compared to plant transpiration requirements. Other studies have indicated that the direct movement of water via extraradical hyphae can be a significant component of a plant’s water uptake, reporting values as high as 12.3% and 17% in alfalfa under high and low water conditions (Wu et al. 2024), 20% in barley (Ruth et al. 2011), or 34.6% in wild oat (Kakouridis et al. 2022). Kakouridis et al. (2022) used dyes and ^18^O to visually demonstrate that water can move directly from *Rhizophagus intraradices* cells across cell membranes and walls to *Avena barbata* cells. The contrasting findings within these studies suggest that the overall contribution of water by AM fungi could be affected by plant-fungus species combinations, substrate type (Pauwels et al. 2023), substrate pore size, root morphology (Allen 2007), and even fungal species (Ruiz-Lozano and Azcón 1995; Augé et al. 2015). It is evident, however, that extraradical hyphae can access and move water within the rhizosphere. In nature, extraradical hyphae are likely to interconnect multiple host plants within CMNs.

Despite our knowledge about the mechanisms of water movement by AM fungi, the partitioning of water among individuals that are interconnected by AM hyphae is not yet resolved (Püschel et al. 2020); even though it is established that CMNs influence whether plant interactions will be competitive or facilitative. We set out to investigate how CMNs comprising a suite of AM fungus species mediate plant interactions among conspecifics under drought and watered conditions in a target plant experiment. To avoid effects on plant physiology and soil moisture due to the presence or absence of AM fungi within treatments, we grew all plants with AM fungi, but manipulated CMN interconnections among plants by keeping CMNs intact or severing them once a week. We chose conspecifics because of their similar rooting depths and resource demands, which would allow us to avoid confounding effects caused by differences in root systems and investigate the role of CMNs beyond that of hydraulic lifting. We hypothesized that if reciprocal rewards influenced water partitioning by CMNs, then we would see competition among target plants and their neighbors, in which large plants with more available C would receive more water in return from CMNs than small individuals. We predicted that this would result in a negative relationship between target and neighbor plant size when they were interconnected by CMNs. We also predicted that drought would decrease plant size due to C limitation, but intact CMNs would increase plant size overall by assisting in water movement among interconnected plants within pots.

## Materials and methods

**Fig. 1:**
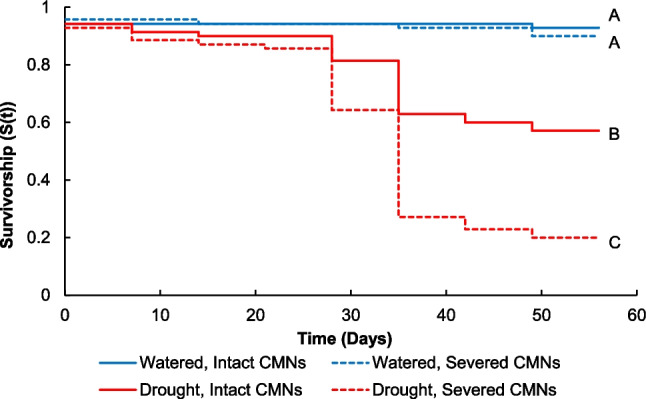
Kaplan–Meier survivorship functions (S(t)) that estimate the probability that an *Andropogon gerardii* individual survived longer than time (t), or 56 days, under watered (blue lines) or drought (red lines) conditions, with intact (solid lines) or severed (dashed lines) common mycorrhizal networks (CMNs). Lines labeled by the same letter do not differ significantly from pairwise comparisons at *P* ≤ 0.0125

In a fully factorial experiment with CMN treatment (intact or severed) and water conditions (watered or drought) as factors with ten replicates in each treatment, forty plastic pots (15.5 cm diameter × 13.5 cm height) were set-up similar to the design of Weremijewicz et al. (2016) and Weremijewicz and Janos (2019; Supplementary Information (SI) Fig. 1a). Seven (1 target plant surrounded by six neighbors) *Andropogon gerardii* Vitman (Everwilde Farms, Sandcreek, WI, USA) individuals were grown in modified Ray Leach cone-tainers (2.5 cm diameter × 12.1 cm length; 49 mL volume; Tangent, OR, USA) that allowed for the establishment of CMNs among individuals. The modified cone-tainers had two layers of fabric covering two, 2 cm × 5 cm slots – first, a nylon silk screen mesh (40 um) pores and then a water-proof Gore-tex (Newwark, DE, USA) layer. Gore-tex has been shown to reduce ion diffusion through soil solution, making it mostly waterproof, but it allows for water vapor to move (Mäder et al. 1993). It is also penetrable by *Funneliformus mosseae* (formerly *Glomus mossae*) hyphae (Mäder et al. 1993) and has been effective in studying the role of CMNs in mineral nutrient partitioning with stable isotopes in previous studies (Weremijewicz et al. 2016). The cone-tainers were equally spaced in each pot (approximately 1.2 cm away from one another), with six cone-tainers surrounding a central target plant within a modified cone-tainer (SI Fig. 1b). Although C4 plants like *A. gerardii*, are often described as being unresponsive to drought (*e.g.* Fay et al. 2003; Chen et al. 2012), we chose *A. gerardii* because of its vertical growth form that precluded aboveground interactions, its relatively high responsiveness to AM fungi (Hetrick et al. 1988; Weremijewicz and Seto 2016), and its success in revealing the effects of CMNs in previous studies (*e.g.* Weremijewicz and Janos 2013).

We filled cone-tainers with a homogenized sandy soil mixture of three parts New Plant Life All Purpose Topsoil (Markman Peat Corp, Le Claire, IA, USA) to one part vermiculite (PVP Industries Inc., North Bloomfield, OH, USA) for some water retention. We limited nutrients to cone-tainers, and thus filled interstices around the cone-tainers with a 4:1 nutrient-poor fine crushed glass (20–40 grade, Harsco Corporation, Camp Hill, PA, USA) and glass bead (12–20 grade, Industrial Supply, Inc, Twin Falls, ID, USA) mixture. The nutrient concentrations of both media can be found in SI Table 1).

We germinated *A. gerardii* seeds on moist paper towels and transplanted seedlings directly into cone-tainers to ensure one seedling per cone-tainer. To facilitate mycorrhiza formation, we added 0.3 ± 0.02 g (SE) of Mycobloom (Kansas, USA) AM fungus inoculum (30 spores cm^−3^ in 70% calcined clay and 30% fine sand, sterilize soil, and other debris (Koziol 2023) into the planting hole within each cone-tainer before transplanting. This inoculum consisted of fungi from seven different fungal species and from six fungal genera isolated from regional prairies (Koziol 2023): *Claroideoglomus claroideum* (N.C. Schenck & G.S. Sm.) Schüssler and Walker 2010, *Funneliformus mosseae* (T.H. Nicolson & Gerd.) Schüssler & Walker 2010, *Cetraspora pellucida* (T.H. Nicolson & N.C. Schenck) Oehl et al. 2008*, Claroideoglomus lamellosum* (Dalpe, Koske & Tews) Schüssler and Walker 2010*, Acaulospora spinosa* C. Walker and Trappe 1981*, Racocetra fulgida* (Koske & C. Walker) Oehl et al. 2008, and *Entrophospora infrequens* (I.R. Hall) R.N. Ames & R.W. Schneid., 1979 in [Błaszkowski et al. 2022].

To facilitate the establishment of CMNs among cone-tainers in pots, seedlings were grown for five weeks without disturbance and equal, ample watering (via misting, three times a day for one hour each). CMN interconnections among neighboring plants likely established in our pot systems because: 1) we used the same experimental setup (including same plant species, as well as the dimensions and makeup of the pots and modified cone-tainers) to that of Weremijewicz et al. (2016) which demonstrated CMNs using stable isotope tracing, 2) a 35 day pre-treatment of ample watering fostered hyphal growth and colonization, 3) cone-tainers of neighboring individuals were 1.2 cm away from one another, which meant that AM hyphal growth rates of 1–4 mm d^−1^ (Jakobsen et al. 1992; Smith et al. 2000; Jansa et al. 2003; Thonar et al. 2011) were highly likely to encounter the root systems of neighboring individuals after 119 days of possible growth within the pots, and 4) the intact CMNs treatments had increased AM colonization rates, suggesting extraradical hyphae reaching from neighboring containers improved mycorrhiza formation.

We imposed treatments of either maintaining CMNs (intact CMNs) or severing them (severed CMNs) under both watering conditions. CMNs were either left intact by not disturbing the cone-tainers throughout the experiment or severed by rotating every cone-tainer within a pot 360° once a week. In previous experiments, rotating cone-tainers weekly was similar to the effects of a solid plastic barrier on plants (Weremijewicz and Janos 2013; Weremijewicz et al. 2016), in which plants from both treatments grew similar in size, had similar effects on foliar nutrition, and competitive interactions among individuals were not detected. After rotation, all pots were watered to eliminate air gaps between cone-tainers and interstitial substrate. To define “drought” conditions in our experiment, field capacity and hygroscopic points of cone-tainer soil and interstitial substrate were quantified following the methods of Wesseling et al. (1957). We maintained cone-tainer soil moisture at 19.7 ± 0.4% (SE) and interstitial substrate moisture at 13.9 ± 0.2% in the drought treatment. In the watered treatment, soil and interstitial substrate moisture were maintained at 26.3 ± 0.3% and 16.1 ± 0.2%, respectively. Soil moisture of every target plant and a neighbor chosen at random was measured every 2–3 days on average using a LabQuest2 Vernier Data Logger and soil moisture sensor (Beaverton, OR, USA). Pots were hand watered to achieve the appropriate soil moisture conditions. Greenhouse temperatures were 21—25 ^◦^ C during the day, and 17—24 ^◦^ C at night. Pot locations were randomized on greenhouse benches to homogenize light levels every other week throughout the pre-treatment and experiment.

After 56 days of treatment, we harvested plants to measure plant dry weights (DWs) and mycorrhizal colonization rates. To collect shoot DWs, we clipped shoots just above the basal meristem and dried them to a constant mass at 60 ^o^ C for three days. We carefully removed roots from each cone-tainer, washed them on a 1-mm sieve, and placed them in 65% ethanol for preservation until the completion of harvest. We then blotted the roots dry, measured their fresh weight, randomly clipped and preserved root clippings in 65% ethanol for assessment of mycorrhizal colonization, and reweighed the root systems. We dried the remaining roots to a constant weight at 60 ^o^ C for three days and reweighed. Using the ratio between fresh weights of pre-and post-clipped roots with the post-clipped DWs, we calculated the DWs of each whole root system.

After weighing *A. gerardii* tissues individually, it was determined that they were too small to meet detection limits for nutrient analysis on shoots, which required 0.25 g of dried tissue, while target and neighbor individuals averaged 0.019 ± 0.005 g (95% CI); therefore, we grouped tissues of similarly sized individuals within their respective treatments. This grouping process went as follows: separately for neighbor and target plants, we rank ordered shoot DWs of all surviving plants within each treatment. We then divided the individual plants into groups that totaled approximately 0.25 g of dried shoot tissue, resulting in similarly sized plants within each group. Each treatment had a different number of pooled samples because treatments with larger plants and better survival met the minimum tissue requirement with fewer plants than treatments with low survival and small individuals (N: Watered, Intact CMNs = 15; Watered, Severed CMNs = 11; Drought, Intact CMNs = 8; Drought, Severed CMNs = 3). Nutrient concentrations of each group were quantified with a nitric perchloric digest followed by ICP spectroscopy at Kansas State Agronomy Soil Testing Laboratory (Manhattan, KS, USA). We investigated the effects of treatment on the concentrations of nutrients that have been associated with AM fungi, like P (Smith and Read 2010), N (He and Dijkstra 2014), Zn (Jansa et al. 2003), Mn, Fe, and Cu (Lehmann and Rillig 2015). These nutrients can play an important role in physiological and biochemical processes like photosynthesis, enzymatic metabolism, reproduction, and defense against pathogens (Moreno Jiménez et al. 2024), and these processes are affected by drought.

Using the same groupings of individuals as for nutrient analyses, we composited root clippings and assessed the AM colonization rate. We cleared roots in 10% KOH at room temperature for 7 days, acidified them in 5% HCl for 30 min, and then placed them in 0.05% Trypan blue in lactoglycerol for 24 h at room temperature to stain AM fungi. For each group, we mounted thirty 1–2 cm fragments and quantified mycorrhizal colonization of roots using a magnified intersection method at 100 x (McGonigle et al. 1990), examining approximately 187 ± 30 (mean ± SD) intersections per group.

### Statistical methods

To assess survival time of all plants (both targets and neighbors) in the different treatments, we calculated Kaplan–Meier survivorship functions (S(t)), or the probability than an individual survives longer than time (t), which was 56 days for this experiment. We made four total pairwise comparisons of survivorship distributions between each treatment using Logrank tests and Bonferroni corrected our value of significance for the multiple statistical tests (α = 0.0125).

Plant growth was measured using whole plant, aboveground, and belowground DWs, as well as root-to-shoot ratios of both target and neighbor plants, which were averaged per pot (the unit of replication). By harvest, the average number of total individuals per pot was the following for each treatment: Watered, Intact CMNs = 6.6 ± 0.4 (95% CI) plants; Watered, Severed CMNs = 6.8 ± 0.4 plants; Drought, Intact CMNs = 5.7 ± 1.1; Drought, Severed CMNs = 5.4 ± 0.6. We assessed the effects of CMNs (intact or severed), watering conditions (watered or drought), and their interaction using per-pot values and two-factor ANOVAs. We tested for the assumption of normal residuals prior to conducting two-factor ANOVAs using Shapiro–Wilk tests and for the assumption of homogenous variance using Levene’s Tests. We found that aboveground DW residuals violated normality due to one outlier – a pot in the Watered, Intact CMNs treatment. This outlier was removed according to Chauvenet’s Criterion (Taylor 1982), which uses the probability of obtaining values as high as those observed from each potential outlier relative to the SD for its treatment. The eliminated pot had less than a 5% chance of having values as high as observed relative to all others in their treatment, and its value misrepresented its treatment. The removal of this outlier slightly decreased the mean DW for the watered, intact CMNs treatment (SI Table 2) but did not affect the statistical results (SI Table 3). We then reconducted the two-factor ANOVA. Root-to-shoot ratio residuals also violated normality, but did not have any outliers, so we conducted ANOVAs on the log transformed root-to-shoot ratio data. We compared differences among treatments using Least Significant Difference (LSD) *post hoc* tests.

We examined the data for evidence of plant interactions, such as competition or facilitation, using Principal Component Analyses (PCAs). Competition and facilitation are revealed as either a negative or positive correlations, respectively, between central target and neighbor plant sizes within pots. To summarize the patterns in covariation among shoot DWs within pots, we performed PCAs for each treatment similar to the methods of Weremijewicz and Janos (2013). Briefly, within each pot, neighbor shoot DWs were rank ordered by size into six rank categories ranging from the first largest neighbor to the smallest neighbor. If a neighbor category resulted in more than 15% zeros (dead plants), the category was then eliminated from the analysis. This approach resulted in four total neighbor rank categories (out of six) in this experiment. Two pots in the watered treatment with severed CMNs and one pot in the drought treatment with severed CMNs were excluded from this analysis because the central, target plant had died prior to harvest. PCA axes were derived from variance/covariance cross-product matrices and Principal Component Axis 1 summarized the variance in neighbor plant sizes (4 neighbors per pot), while Axis 2 plotted target plant size. After assessing the significance of the axes, we rotated Axis 1 to be congruent with the third largest neighbor to create the strongest Pearson correlations between all neighbors with Axis 1 (Pearson correlations before rotation are provided in SI Table 4). We then calculated Pearson correlations between the target shoot DWs and Axis 1. This process was repeated for each treatment.

We examined shoot nutrient concentrations and AM colonization rates for the effect of watering conditions, CMNs, and their interaction using two-way ANOVAs and LSD post hoc tests after testing for assumptions. The residuals for S, Cu, Fe, Mn, and Zn concentrations did not meet normality. For S, Cu, and Zn concentrations there was a single outlier from different groups, whose residuals did not meet Chauvenet’s Criterion, so they were removed prior to further analysis. We additionally had to log transform the S, Fe, Mn, and Zn concentration data to meet normality, while for Cu concentrations, the data were square root transformed. Colonization rate data residuals were non-normal as well, but removing one outlier, from the Intact CMNs, Watered treatment normalized them. To analyze the data for a relationship between root length colonized and whole plant DW, root length colonized and shoot P concentrations, we used least-squares linear regressions for each treatment using data that only had normal residuals (for example, if an outlier caused residuals to be non-normal when conducting two-way ANOVAs, that outlier remained omitted for linear regressions).

Survival analyses, least square linear regressions and ANOVAs were conducted in Statistix 10 (Tallahassee, FL). Principal component analyses were conducted using PC-ORD v.7 (McCune and Mefford 2011). Statistical significance, unless otherwise noted, was determined at α = 0.05.

## Results

We found that survivorship was improved by watering and intact CMNs (Fig. 1). Overall, drought decreased survivorship for plants with intact CMNs (L = −4.73, *P* < 0.0001) and severed CMNs (L = −8.02, *P* < 0.0001) compared to watered conditions. Within drought treatments, plants with intact CMNs had increased survivorship compared to plants with severed CMNs (L = 4.29, *P* < 0.0001). When watered, plants with intact and severed CMNs did not have differences in survivorship (L = 0.59, *P* = 0.5549).

**Fig. 2:**
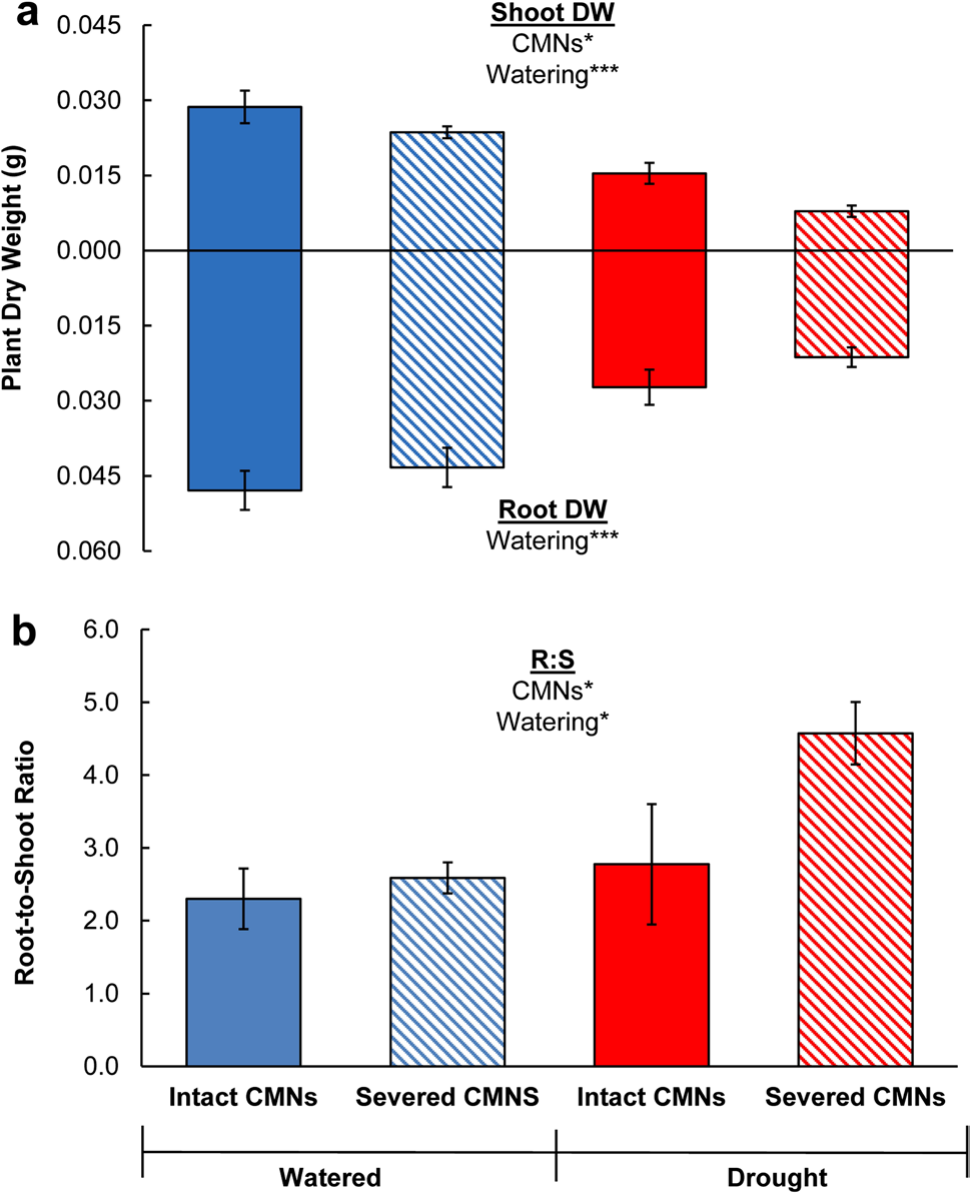
Mean dry weights (g) of aboveground (shown as values above the horizontal access) and belowground (shown as positive values below the horizontal axis) tissues (**a**) and root to shoot ratios (**b**) of averaged across both target and neighbor *Andropogon gerardii* individuals within pots grown in a target plant experiment under watered (blue bars) or drought (red bars) conditions, with intact (solid bars) or severed (hashed bars) common mycorrhizal networks (CMNs). Error bars indicate standard error. Two-way ANOVA results reported as * *P* < 0.05, ** *P* < 0.01, and *** *P* < 0.001

Plant growth was improved by both CMNs and watering, but not by the interaction of the two factors (Table 1). Average shoot, root, and whole plant DWs per pot were larger under watered conditions than drought conditions (Fig. 2a). Plants with intact CMNs had larger shoot and whole plant DWs compared to plants with severed CMNs (Fig. 2a). Average root-to-shoot ratios per pot were also affected by both CMNs and watering (Table 1). Plants with severed CMNs had larger root-to-shoot ratios than those with intact CMNs, and plants under drought had larger ratios than those in watered conditions (Fig. 2b), thus, plants with severed CMNs under drought conditions had the largest root-to-shoot ratios of all (Fig. 2b).

**Table 1:** Two-way ANOVA main effects and interactions results for *Andropogon gerardii* individuals averaged per pot (*df* = 1, 36) and grown under two different watering conditions (watered or drought) and two different common mycorrhizal network (CMN) treatments (intact or severed)

Dependent	Factor	*F*	*P*
Whole Plant DW	CMNs	4.19	0.0487
	Watering	49.13	< 0.0001
	Watering x CMNs	0.28	0.6001
Shoot DW	CMNs	7.23	0.0113
	Watering	64.51	< 0.0001
	Watering x CMNs	3.26	0.0806
Root DW	CMNs	1.60	0.2141
	Watering	34.76	< 0.0001
	Watering x CMNs	0.10	0.7581
Root-to-Shoot	CMNs	4.24	0.0475
	Watering	5.13	0.0301
	Watering x CMNs	1.91	0.1758

**Fig. 3:**
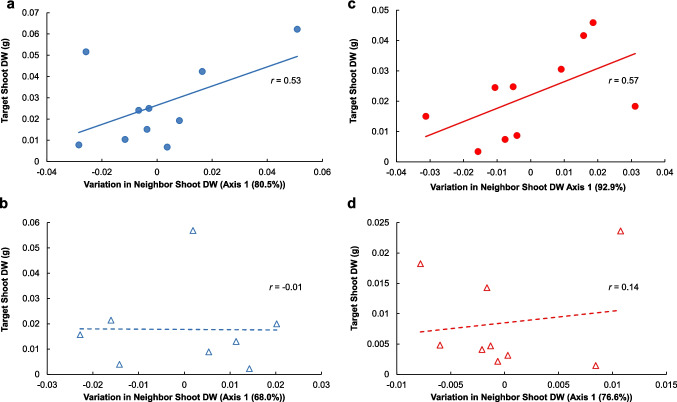
Target plant sizes per pot based on shoot dry weights (g; DWs) of *Andropogon gerardii* overlayed onto Principal Component Axis 1, which was derived from variance/covariance cross-product matrices and significantly (*P* < 0.05) summarized the variation in neighbor (four per pot) shoot dry weights. The axis was rotated to be congruent with the third largest neighbor, which created the strongest Pearson correlations between all neighbors with Axis 1. Pearson's correlation coefficients with Axis 1 (*r*) are reported for each treatment: watered (**a**, **b**) or drought conditions (**c**, **d**), with intact (**a**, **c**) or severed (**b**, **d**) common mycorrhizal networks (CMNs). The positive correlations between target and neighbor plant sizes in treatments with intact CMNs (**a**, **c**) indicate positive, potentially facilitative interactions within pots, while the relatively weaker correlations in treatments with severed CMNs treatments (**b**, **d**) indicate little to no plant interactions

Principal Component Analyses revealed that regardless of watering treatment, and only when CMNs were intact, target plant size was positively associated with neighbor size variance. When CMNs were severed, however, there was no such association between target and neighbor plant sizes (Fig. 3). The first PCA axis represented the majority of the variance among neighboring plants for all four treatments and no other axes were significant (Table 2). Rotation of PCA Axis 1 to be congruent with the third largest neighbor provided the most correlation with the axis for all four neighbors across all four treatments overall (Table 2). For the intact CMNs treatments, the largest and second largest neighbors were more congruent with this axis after rotation, while for the severed CMNs, it was the fourth largest neighbor that was most strongly congruent with this axis.

**Table 2:** Principal components analysis (PCA) percentage of variance represented by the first axis, loadings on Axis 1 for the four PCA-summarized neighbor categories, and the correlation between Axis 1 and target shoot dry weights for treatments with drought or watered conditions and with severed or intact common mycorrhizal networks (CMNs). Axis 1 was rotated to be congruent with the third largest neighbor to create the strongest Pearson correlations between all neighbors with Axis 1. For all treatments, the first axis was the only significant axis in explaining the variance among neighboring plants

	Percentage of variance (P*)	Pearson's correlation coefficient with Axis 1^†^, *r*				
Treatment	Principal Component Axis 1	Largest neighbor	2nd largest neighbor	3rd largest neighbor	4th largest neighbor	Targets
Watered, Intact CMNs	80.5% (0.001)	0.878	0.957	0.922	0.801	0.530
Watered, Severed CMNs	68.0% (0.011)	−0.780	−0.036	0.983	0.960	−0.01
Drought, Intact CMNs	92.9% (0.001)	0.951	0.986	0.987	0.904	0.567
Drought, Severed CMNs	76.6% (0.005)	0.564	0.714	0.945	0.949	0.144

*The probability is from a randomization test with 999 runs

†Axis 1 was rotated to maximize congruence with the largest, nearest neighbor for each PCA

**Fig. 4:**
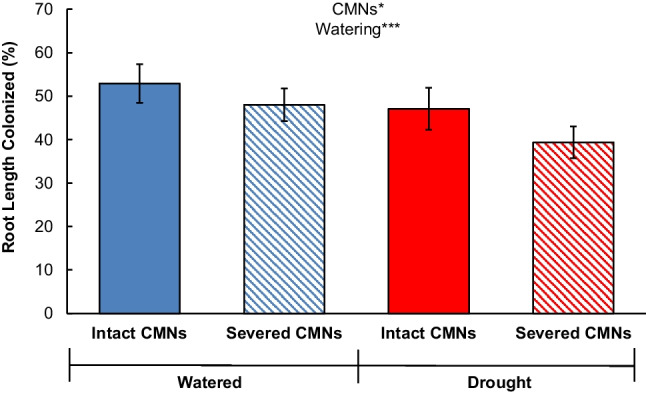
Mean arbuscular mycorrhizal colonization rates (%) of *Andropogon gerardii* root length in watered (blue bars) or drought (red bars) treatments, with intact (solid bars) or severed (hashed bars) common mycorrhizal networks (CMNs). Error bars indicate standard error. Two-way ANOVA results reported as * *P* < 0.05, ** *P* < 0.01, and *** *P* < 0.001

**Fig. 5:**
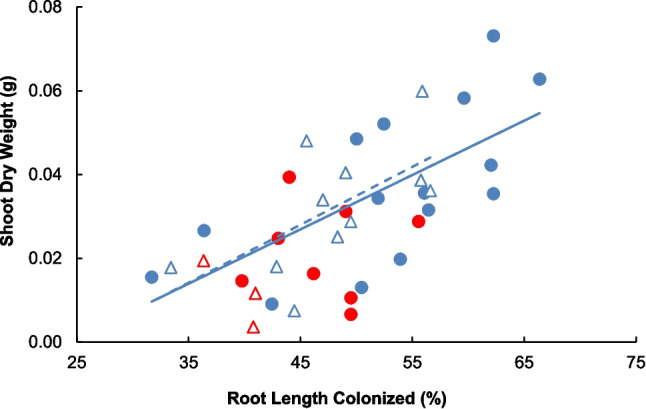
The relationship between mean shoot dry weight (g) and arbuscular mycorrhizal colonization rate (%) of *Andropogon gerardii* roots when plants were grown under watered (blue symbols) or drought (red symbols) conditions, with intact (solid lines, circles) or severed (dashed lines, triangles) common mycorrhizal networks (CMNs). Regression lines are shown only for significant linear regressions, which were for both CMN treatments in the watered conditions

Both intact CMNs and watering resulted in increased mycorrhizal colonization rates (Fig. 4; CMNs: *F*_1,32_ = 6.81; *P* = 0.0136; Watering: *F*_1,32_ = 8.77; *P* = 0.0057) with no interaction between the two factors (*F*_1,32_ = 0.06; *P* = 0.8149). Increasing rates of AM colonization of roots (RLC; %) increased shoot growth in watered conditions for both CMN treatments (Fig. 5; Intact CMNs: *F*_1,12_ = 8.07, *P* = 0.0149, Shoot DW = 0.0015 (RLC) – 0.0403; Severed CMNs: *F*_1,9_ = 5.84, *P* = 0.0389, Shoot DW = 0.0014 (RLC) – 0.0410). Neither the slopes (*F*_1,21_ = 0.01, *P* = 0.9300), nor the constants (*F*_1,22_ = 0.19, *P* = 0.6655), between severed and intact CMNs under watered conditions differed from one another. There was no relationship between RLC and plant size under drought (Intact CMNs: *F*_1,6_ = 0.00 *P* = 0.9788; Severed CMNs: *F*_1,2_ = 2.4 *P* = 0.3650).

**Fig. 6:**
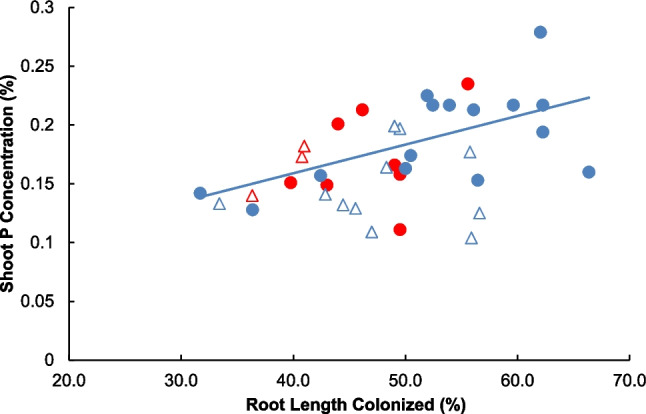
The relationship between shoot phosphorus concentrations (%) vs. total root length colonized by arbuscular mycorrhizal fungi (%) of *Andropogon gerardii* seedlings grown under watered (blue symbols) or drought (red symbols) conditions, with intact (circles) or severed (triangles) common mycorrhizal networks (CMNs). Linear regressions were significant only for plants with intact CMNs under watered conditions (indicated by a solid blue line)

Shoot nutrient concentrations of Ca, S, Fe, and Mn were affected by watering (SI Table 5), and plants under drought had higher levels of Ca, S, Fe, and Mn. CMNs affected Fe and Zn concentrations, and plants with severed CMNs had higher Fe concentrations, while those with intact CMNs had higher Zn concentrations. An interaction between CMNs and watering was found for S, Fe, and Zn (SI Table 5). Although average P concentrations were not affected by CMNs nor watering (SI Table 5), P concentrations increased with AM colonization rates, only when plants had intact CMNs under watered conditions (Fig. 6; *F*_1,12_ = 6.95, *P* = 0.0205, Shoot P = 0.0024 (RLC) + 0.0613; Severed CMNs, Watered: *F*_1,9_ = 0.08, *P* = 0.7849; Intact CMNs, Drought:* F*_1,6_ = 0.76, *P* = 0.4157; Severed CMNs, Drought: *F*_1,2_ = 35.07, *P* = 0.1065).

## Discussion

In our study, all plants were colonized by AM fungi, but only plants with intact CMNs benefited in both survival and growth. Contrary to our hypothesis, however, the partitioning of water by CMNs did not intensify competitive interactions between targets and neighbors. Despite the pressure of being surrounded by six individuals within a pot, by the end of the experiment, large targets were surrounded by large, rather than small, neighbors, indicating facilitative interactions were mediated by CMNs. Plants with intact CMNs had improved colonization rates, suggesting that extraradical hyphae improved mycorrhiza formation in neighboring root systems. These additional hyphae likely provided pathways for enhanced water and nutrient movement within pots, which would have benefited the growth of neighboring individuals. Watering also improved AM colonization, which increased plant growth, but a positive relationship between colonization and shoot P concentration only existed when CMNs were intact. Under drought conditions, plants most likely derived some other benefit from the AM symbiosis. Because most nutrients other than N were examined, the Law of the Minimum (von Liebig 1840) would suggest that either water, N, or both were limiting growth and/or survival in this treatment. Under drought conditions, mycorrhizal fungi can acquire significant amounts of N for their hosts, particularly from inorganic sources dissolved within soil solution (Püschel et al. 2023). In our experiment, either of these possible limitations were severe enough to considerably affect survival, and extraradical hyphae of CMNs likely alleviated these limitations.

In plants and in soil surrounding roots, water moves passively from high to low water potentials, driven by transpiration. For example, hydraulic lifting by roots moves water from deep, saturated soil profiles towards shallower, water-limited ones via root systems, where it is redistributed (Allen 2022), particularly by CMNs to shallow-rooted individuals (Egerton-Warburton et al. 2007, 2008; Saharan et al. 2018; Singh et al. 2019, 2020), resulting in facilitative interactions between differently rooted species. In our study, though, conspecific individuals did not have such large differences in root system size, but facilitative interactions still developed among interconnected plants within pots. Factors that improved the passive movement of water, like increased hydraulic conductivity and decreased resistance due to the increased presence of extraradical hyphae in the soil (Augé et al. 2001, 2007; Bitterlich et al. 2018; Pauwels et al. 2023), likely caused plants in the intact CMNs treatment to benefit. Because we did not find evidence for competitive interactions, reciprocal rewards were likely not at play in our experiment. Rather than dominating limiting resources via CMNs through C provisioning, C transfer by large plants likely fostered AM fungus hyphae instead, benefiting neighboring individuals with improved colonization rates and ultimately plant size and survival.

An increase in the rate of AM colonization with watering and intact CMNs likely improved the transport capacity of water from hyphae to plants, resulting in larger growth. Increased rates of root colonization are associated with improved stomatal conductance rates, by about 46% on average (Augé et al. 2015). Soil colonization, like that by extraradical hyphal networks, can affect soil water retention abilities (Augé et al. 2001) and positively affect stomatal conductance (Augé et al. 2007). When plants were interconnected in CMNs within pots, the extraradical hyphae could both directly and indirectly provide water to hosts, such as by moving water directly to plants within hyphae (Kakouridis et al. 2022), altering the geometry of water movement within the soil matrices (Püschel et al. 2021), and maintaining moisture for longer in drying soils (Augé et al. 2001). In contrast, plants with severed CMNs had hyphae constrained to the resources within their respective cone-tainers. To assess for the potential effect of extraradical hyphae on soil moisture, we examined the rate of drying (% d^−1^) within either cone-tainer soil or interstitial substrate over the course of the experiment, but no main effects of CMNs were detected (SI Table 6). This result is most likely because our watering regime regularly brought all of the treatments, regardless of CMNs, back up to a designated level of substrate moisture, negating the ability for us to detect any potential subtle effects of extraradical hyphae on water holding capacity of substrates. Root colonization was decreased when CMNs were severed, and no relationships were found between colonization and plant size. Although we did not measure stomatal conductance in this experiment because of the initial focus on ecological, rather than physiological, consequences of CMNs, the increased root-to-shoot ratios of plants under drought stress, especially with severed CMNs, indicated a morphological response, most likely in search of water (Taiz et al. 2015).

Water is likely not exchanged nor controlled by C provisioning by hosts because of the passive nature of water uptake by plants at the cellular level. The presence of AM fungi is known to enhance aquaporin expression in host plants, improving the permeability of water into hosts (Uehlein et al. 2007; Quiroga et al. 2019). At the periarbuscular membrane, aquaporins move water from fungus to plant via facilitated diffusion (Uehlein et al. 2007; Quiroga et al. 2019). In contrast, for example, nutrient transporters like H^+^/Pi symporters involve the active transport of nutrients (Dreyer et al. 2019; Wipf et al. 2019), and fungi have the capability to control the amount of P delivered to the host by either reabsorbing inorganic P or leaving it in the periarbuscular space (Balestrini et al. 2007; Walder et al. 2016; Wipf et al. 2019). Such mechanisms have not been demonstrated for water movement across aquaporins. CMN-mediated water movement, therefore, may not be under metabolic control, which may mean that CMN-mediated plant interactions for water cannot be competitive.

The outcome of CMN-mediated plant interactions is likely to be influenced by the watering regime of an experiment because it ultimately influences the most growth-limiting resource. In previous, similar experiments, *A. gerardii* individuals were well watered and CMNs consistently mediated competitive, not facilitative, interactions (Weremijewicz and Janos 2013; Weremijewicz et al. 2016, 2018). Singh et al. (2020) demonstrated that although hyphal networks can mediate facilitative interactions through hydraulic lifting under drought conditions, competitive interactions for mineral nutrients emerged among interconnected plants in well-watered conditions. Under drought stress, plants close stomata to minimize water loss through transpiration, limiting access to CO_2_ (Taiz et al. 2015), which may stunt growth due to C-limitation. In contrast, well-watered conditions increase plant access to C, causing growth to increase but also to become limited by mineral nutrients, which may induce competition among plants. CMNs may then mediate competition among individuals by partitioning limiting mineral nutrients to hosts with different photosynthate provisioning abilities. In previous experiments, *A. gerardii* plants were watered regularly to saturation to promote growth (Weremijewicz and Janos 2013; Weremijewicz et al. 2016, 2018). In this experiment, however, we created long-term drought conditions, and it is likely that in reality, our watered treatment may have been a moderate drought treatment. In the watered treatment, plants were watered to field capacity which would have caused soils to desiccate between waterings more quickly than if we were to water to saturation. In between waterings, the macropores (> 80 μm) of the substrates would have desiccated first, concentrating water and dissolved mineral nutrients to micropores (< 30 μm) that were inaccessible to roots and root hairs, but were accessible to hyphal networks (Allen 2022).

In the watered treatment, hyphal networks likely increased access to water and P simultaneously. It is well established that the simple presence of AM fungi can improve drought avoidance of host plants by relieving P stress (Augé 2001), but Püschel et al. (2021) demonstrated that external AM hyphae are significant for intermediate-term P uptake during moderate droughts. Water found within soil micropores is important for plants because dissolved anionic nutrients, like P, become concentrated (Allen 2007), and AM hyphae can move this P directly to their hosts when under moderate drought stress (Püschel et al. 2021). We found that as root colonization by AM fungi increased, so did shoot P concentrations, but only for plants with intact CMNs in the watered treatment. In this treatment, the additional water access may have likely caused stomates to open, which would begin photosynthesis and create a demand for nutrients like P, which hyphal networks were able to access. This improved access to resources from intact CMNs likely contributed to improved growth and survival.

In the severe drought treatment, intact CMNs increased survival, plant growth, and root colonization, and mediated facilitative plant interactions. Unlike in the watered treatment, however, a lack of relationships among variables makes the mechanisms behind these results less clear. Mineral nutrient analyses revealed that plants under drought stress built-up high levels of micronutrients, and few CMN effects were found. In fact, when CMNs had a statistically significant effect on nutrient levels, plants with severed CMNs had the highest concentrations, suggesting the most C-stressed individuals accumulated mineral nutrients, most likely because they were unable to allocate them towards growth. The lack of a relationship between AM colonization rates and plant size for both CMN treatments under drought stress may indicate a drought effect on the AM fungi themselves. In general, AM fungi may decrease sporulation rates and increase root colonization when water stressed (Augé, 2001). In our study, though, overall colonization rates were decreased under drought conditions, consistent with the findings of Püschel et al. (2023). Leyva-Morales et al. (2019) found that *Rhizophagus intraradices* decreases the surface area exposed to drought by decreasing fine hyphae and increasing the production of large-diameter hyphae instead. Additionally, plants have been shown to shunt C away from some fungus species that were present in our inoculum. For example, under drought stress, *C. claroideum* decreases colonization rates (Forczek et al. 2022; Geneva et al. 2022). On the other hand, *F. mosseae* may be beneficial to plants under drought conditions (Forczek et al. 2022; Lidoy et al. 2023), and *C. pellucida* is present in semi-arid tropical dry forests in Brazil (Pagano et al. 2013). Another possible explanation for the decrease in colonization rates under drought conditions is an increase in root production by hosts. Colonization rates are similar to concentration measurements by being a measurement of quantity per area. An increase in root production (as indicated by root-to-shoot ratios) by plants under drought may have “diluted” colonization rates for plants in this treatment. It can also take weeks to reach peak colonization by AM fungi (Graham et al. 1991), which may mean that colonization had not yet caught up with root production. Regardless of the potential effects of soil drying on AM hyphae, the presence of intact CMNs improved colonization of roots overall, and this increased access to CMNs likely fostered positive, facilitative plant interactions among target plants and neighbors.

Before plants can interact with one another, they must first survive. Because all treatments included AM colonization in our study, our findings suggest that it is not just the simple presence of AM fungi within plant roots that can positively influence aspects of plant fitness such as survival and growth, but rather the networks of extraradical AM hyphae. The fitness benefits of AM fungi are often described as nutritive, but our study supports Delavaux et al.’s (2017) findings that non-nutritive benefits, like water access, can also be significant. In our experiment, all plants were colonized by AM fungi, but under drought conditions, survivorship increased three-fold for plants with intact CMNs. In a similar system, Weremijewicz et al. (2018) also found that CMNs improved survival of *A. gerardii* and *Elymus canadensis*. Carbon isotope analyses, that were only possible on C3 *E. canadensis*, revealed that CMNs improved water uptake, which increased stomatal opening and access to atmospheric CO_2_, altering its carbon isotopic signature. Apart from this study, however, few have reported the effects of CMNs on survival. Generally, there is a demand for tissue samples for post-harvest nutrient analyses, thus, imposed periods of drought purposefully prevent mortality. In fact, Gehring et al. (2017) suggested that most nonagricultural studies investigating AM fungi are too short in duration to understand the impacts of drought. In our experiment, plants experienced drought for 8 weeks, while in other greenhouse studies, droughts range from four days (Püschel et al. 2020) to five weeks (Püschel et al. 2021), while in the field, drought can be as long as twelve weeks (Miller et al. 1995).

In nature, although plants interact via both CMNs and roots, as well as experience diffusion of water and mineral nutrients with rainfall, our model system was necessary to elucidate the specific role of CMNs under drought conditions. Although it is possible that the mechanical disruption of soil from the rotation of cone-tainers to sever networks may have had an effect in our experiment, previous experiments with the same experiment design but with a third treatment using solid, undisturbed cone-tainers to test for this artifact, have found that this treatment is statistically identical to the severed CMNs treatments (Weremijewicz and Janos 2013; Weremijewicz et al. 2016). We additionally excluded a sterile control from our experiment design because 1) AM fungi have strong physiological effects on plants that affect drought tolerance, and 2) we sought to examine the role of interconnecting, extraradical hyphae on plant interactions, not just the presence/absence of AM fungi. Additionally, *A. gerardii* is relatively dependent upon and highly responsive to AM fungi (sensu Janos 2007). Based on the outcomes of previous work, it likely would have grown poorly without AM fungi (Hartnett et al. 1993; Weremijewicz and Seto 2016), which would preclude it from interacting with neighbors (Hartnett et al. 1993). Our system also included a suite of AM fungus species, which may be necessary to reveal the role of CMNs under drought because the interactions between plants and fungi within CMNs are dynamic, with differences in functional traits and benefits received from different fungus species (Kiers et al. 2011; Thonar et al. 2011; Walder and van der Heijden 2015) and shifts in carbon allocation by hosts to beneficial fungus species under drought, like *F. mosseae* (Forczek et al. 2022). Although it is also possible that plants may have interacted for additional N from both organic and inorganic sources within the substrates (Püschel et al. 2023), we were unable to measure this nutrient due to insufficient plant tissue quantities. Regardless, it has been suggested that the P uptake by plants could be more compromised than N uptake under drought conditions (He and Dijkstra 2014; Püschel et al. 2021).

In conclusion, CMNs interconnecting large plants with their neighbors promote plant survival and growth during periods of drought. Although caution must be taken when extrapolating results from carefully controlled greenhouse experiments, the results of our experiment may relate to some patterns seen in the field. For example, increased external AM hyphal production can aid the recovery of prairie plant communities after drought (Miller et al. 1995). This phenomenon may also explain why facilitative interactions across CMNs have been found in arid environments (Montesinos-Navarro et al. 2016). Although our findings are in contrast to many others that have found CMNs increase competition, one result has remained consistent – connection to CMNs, in general, benefits plant growth overall. Whether plants experience competition or facilitation, however, is likely to depend upon the limiting resource under the environmental conditions at the time of observation. If the effects of water stress can influence future generations, as (Puy et al. 2022) suggests, then by improving plant fitness, CMNs have important implications for plant interactions in a changing world.

## Supplementary Information

Below is the link to the electronic supplementary material.

Supplementary file1 (DOCX 787 KB)

## Data Availability

All data are presented in the manuscript as tables and figures. Data are available from Open Science Framework at osf.io/nqegz.

## References

[CR1] Allen MF (2007) Mycorrhizal fungi: highways for water and nutrients in arid soils. Vadose Zone Journal 6:291–297

[CR2] Allen MF (2022) Mycorrhizal dynamics in ecological systems. Cambridge University Press, United Kingdom

[CR3] Augé RM (2001) Water relations, drought and vesicular-arbuscular mycorrhizal symbiosis. Mycorrhiza 11:3–42

[CR4] Augé RM (2004) Arbuscular mycorrhizae and soil/plant water relations. Can J Soil Sci 84:373–381

[CR5] Augé RM, Stodola AJ, Tims JE, Saxton AM (2001) Moisture retention properties of a mycorrhizal soil. Plant Soil 230:87–97

[CR6] Augé RM, Toler HD, Moore JL et al (2007) Comparing contributions of soil versus root colonization to variations in stomatal behavior and soil drying in mycorrhizal *Sorghum bicolor* and *Cucurbita pepo*. J Plant Physiol 164:1289–1299 17189660 10.1016/j.jplph.2006.08.005

[CR7] Augé RM, Toler HD, Saxton AM (2015) Arbuscular mycorrhizal symbiosis alters stomatal conductance of host plants more under drought than under amply watered conditions: a meta-analysis. Mycorrhiza 25:13–24 24831020 10.1007/s00572-014-0585-4

[CR8] Balestrini R, Gómez-Ariza J, Lanfranco L, Bonfante P (2007) Laser microdissection reveals that transcripts for five plant and one fungal phosphate transporter genes are contemporaneously present in arbusculated cells. Mol Plant Microbe Interact 20:1055–1062 17849708 10.1094/MPMI-20-9-1055

[CR9] Bárzana G, Aroca R, Bienert GP et al (2014) New insights into the regulation of aquaporins by the arbuscular mycorrhizal symbiosis in maize plants under drought stress and possible implications for plant performance. Mol Plant Microbe Interact 27:349–363 24593244 10.1094/MPMI-09-13-0268-R

[CR10] Bitterlich M, Franken P, Graefe J (2018) Arbuscular mycorrhiza improves substrate hydraulic conductivity in the plant available moisture range under root growth exclusion. Front Plant Sci 9:301 29563923 10.3389/fpls.2018.00301 PMC5845879

[CR11] Błaszkowski J, Sánchez-García M, Niezgoda P et al (2022) A new order, Entrophosporales, and three new Entrophospora species in Glomeromycota. Front Microbiol 13:962856. 10.3389/fmicb.2022.962856 36643412 10.3389/fmicb.2022.962856 PMC9835108

[CR12] Brundrett MC, Tedersoo L (2018) Evolutionary history of mycorrhizal symbioses and global host plant diversity. New Phytol 220:1108–1115 29355963 10.1111/nph.14976

[CR13] Caretta MA, Mukherji A, Arfanuzzaman M, et al (2022) Water. In: Pörtner H-O, Roberts DC, Tignor M, Poloczanska ES, Mintenbeck K, Alegría A, Craig M, Langsdorf S, Löschke S, Möller V, Okem A, Rama B (eds) Climate Change 2022: Impacts, Adaptation, and Vulnerability, Contribution of Working Group II to the Sixth Assessment Report of the Intergovernmental Panel on Climate Change. Cambridge University Press, pp 555–668. 10.1017/9781009325844

[CR14] Chen G, Tian H, Zhang C et al (2012) Drought in the Southern United States over the 20th century: variability and its impacts on terrestrial ecosystem productivity and carbon storage. Clim Change 114:379–397. 10.1007/s10584-012-0410-z

[CR15] Delavaux CS, Smith-Ramesh LM, Kuebbing SE (2017) Beyond nutrients: a meta-analysis of the diverse effects of arbuscular mycorrhizal fungi on plants and soils. Ecology 98:2111–2119 28500779 10.1002/ecy.1892

[CR16] Dreyer I, Spitz O, Kanonenberg K et al (2019) Nutrient exchange in arbuscular mycorrhizal symbiosis from a thermodynamic point of view. New Phytol 222:1043–1053 30565261 10.1111/nph.15646 PMC6667911

[CR17] Duc NH, Csintalan Z, Posta K (2018) Arbuscular mycorrhizal fungi mitigate negative effects of combined drought and heat stress on tomato plants. Plant Physiol Biochem 132:297–307. 10.1016/j.plaphy.2018.09.011 30245343 10.1016/j.plaphy.2018.09.011

[CR18] Egerton-Warburton LM, Querejeta JI, Allen MF (2007) Common mycorrhizal networks provide a potential pathway for the transfer of hydraulically lifted water between plants. J Exp Bot 58:1473–1483. 10.1093/jxb/erm009 17350936 10.1093/jxb/erm009

[CR19] Egerton-Warburton LM, Querejeta JI, Allen MF (2008) Efflux of hydraulically lifted water from mycorrhizal fungal hyphae during imposed drought. Null 3:68–71. 10.4161/psb.3.1.4924 10.4161/psb.3.1.4924 PMC2633966 19704776

[CR20] El-Samad HMA, El-Hakeem KNSA (2019) Strategy Role of Mycorrhiza Inoculation on Osmotic Pressure, Chemical Constituents and Growth Yield of Maize Plant Gown under Drought Stress. Am J Plant Sci 10:1102–1120. 10.4236/ajps.2019.106080

[CR21] Fang Y, Xiong L (2015) General mechanisms of drought response and their application in drought resistance improvement in plants. Cell Mol Life Sci 72:673–689. 10.1007/s00018-014-1767-0 25336153 10.1007/s00018-014-1767-0 PMC11113132

[CR22] Fay PA, Carlisle JD, Knapp AK et al (2003) Productivity responses to altered rainfall patterns in a C4-dominated grassland. Oecologia 137:245–251. 10.1007/s00442-003-1331-3 12845518 10.1007/s00442-003-1331-3

[CR23] Fellbaum CR, Mensah JA, Cloos AJ et al (2014) Fungal nutrient allocation in common mycorrhizal networks is regulated by the carbon source strength of individual host plants. New Phytol 203:646–656. 10.1111/nph.12827 24787049 10.1111/nph.12827

[CR24] Forczek ST, Bukovská P, Püschel D et al (2022) Drought rearranges preferential carbon allocation to arbuscular mycorrhizal community members co-inhabiting roots of *Medicago truncatula*. Environ Exp Bot 199:104897

[CR25] Gehring CA, Swaty RL, Deckert RJ (2017) Chapter 16 - Mycorrhizas, Drought, and Host-Plant Mortality. In: Johnson NC, Gehring C, Jansa J (eds) Mycorrhizal Mediation of Soil. Elsevier, pp 279–298

[CR26] Geneva M, Kirova E, Sichanova M et al (2022) Physiological analysis of drought stress influenced by *Claroideoglomus claroideum* inoculation of in vitro or seed-propagated *Coleus forskohlii* Briq plants. Biologia 78:641–654. 10.1007/s11756-022-01231-3

[CR27] Graham JH, Eissenstat DM, Drouillard DL (1991) On the Relationship Between a Plant’s Mycorrhizal Dependency and Rate of Vesicular-Arbuscular Mycorrhizal Colonization. Funct Ecol 5:773–779. 10.2307/2389540

[CR28] Gutjahr C, Paszkowski U (2013) Multiple control levels of root system remodeling in arbuscular mycorrhizal symbiosis. Front Plant Sci 4:204 23785383 10.3389/fpls.2013.00204 PMC3684781

[CR29] Hammer EC, Pallon J, Wallander H, Olsson PA (2011) Tit for tat? A mycorrhizal fungus accumulates phosphorus under low plant carbon availability. FEMS Microbiol Ecol 76:236–244. 10.1111/j.1574-6941.2011.01043.x 21223336 10.1111/j.1574-6941.2011.01043.x

[CR30] Hartnett DC, Hetrick BAD, Wilson GWT, Gibson DJ (1993) Mycorrhizal Influence on Intra- and Interspecific Neighbour Interactions among Co-Occurring Prairie Grasses. J Ecol 81:787–795. 10.2307/2261676

[CR31] He M, Dijkstra FA (2014) Drought effect on plant nitrogen and phosphorus: a meta-analysis. New Phytol 204:924–931 25130263 10.1111/nph.12952

[CR32] Hetrick BD, Kitt DG, Wilson GT (1988) Mycorrhizal dependence and growth habit of warm-season and cool-season tallgrass prairie plants. Can J Bot 66:1376–1380

[CR33] Horton TR (2015) Mycorrhizal networks. Springer, Dordrecht, The Netherlands

[CR34] Jakobsen I, Abbott LK, Robson AD (1992) External hyphae of vesicular-arbuscular mycorrhizal fungi associated with *Trifolium subterraneum* L. New Phytol 120:371–380. 10.1111/j.1469-8137.1992.tb01077.x

[CR35] Janos DP (2007) Plant responsiveness to mycorrhizas differs from dependence upon mycorrhizas. Mycorrhiza 17:75–91 17216499 10.1007/s00572-006-0094-1

[CR36] Jansa J, Mozafar A, Frossard E (2003) Long-distance transport of P and Zn through the hyphae of an arbuscular mycorrhizal fungus in symbiosis with maize. Agronomie 23:481–488

[CR37] Kakouridis A, Hagen JA, Kan MP et al (2022) Routes to roots: direct evidence of water transport by arbuscular mycorrhizal fungi to host plants. New Phytol 236:210–221. 10.1111/nph.1828 35633108 10.1111/nph.18281 PMC9543596

[CR38] Kiers ET, Duhamel M, Beesetty Y et al (2011) Reciprocal rewards stabilize cooperation in the mycorrhizal symbiosis. Science 333:880–882 21836016 10.1126/science.1208473

[CR39] Koziol L (2023) MycoBloom Mycorrhizae: Native Prairie Endomycorrhizal Fungi. In: FAQ: MycoBloom. http://mycobloom.com/faq.html. Accessed 22 Feb 2023

[CR40] Leake J, Johnson D, Donnelly D et al (2004) Networks of power and influence: the role of mycorrhizal mycelium in controlling plant communities and agroecosystem functioning. Can J Bot 82:1016–1045. 10.1139/b04-060

[CR41] Lehmann A, Rillig MC (2015) Arbuscular mycorrhizal contribution to copper, manganese and iron nutrient concentrations in crops – A meta-analysis. Soil Biol Biochem 81:147–158. 10.1016/j.soilbio.2014.11.013

[CR42] Lekberg Y, Hammer EC, Olsson PA (2010) Plants as resource islands and storage units–adopting the mycocentric view of arbuscular mycorrhizal networks. FEMS Microbiol Ecol 74:336–345 20722732 10.1111/j.1574-6941.2010.00956.x

[CR43] Leyva-Morales R, Gavito ME, Carrillo-Saucedo SM (2019) Morphological and physiological responses of the external mycelium of *Rhizophagus intraradices* to water stress. Mycorrhiza 29:141–147. 10.1007/s00572-019-00880-8 30643987 10.1007/s00572-019-00880-8

[CR44] Lidoy J, López-García Á, Amate C et al (2023) Regulation of mycorrhizal colonization under stress in tomato depends on symbiotic efficiency. Environ Exp Bot 215:105479. 10.1016/j.envexpbot.2023.105479

[CR45] Mäder P, Vierheilig H, Alt M, Wiemken A (1993) Boundaries between soil compartments formed by microporous hydrophobic membranes (GORE-TEX ®) can be crossed by vesicular-arbuscular mycorrhizal fungi but not by ions in the soil solution. Plant Soil 152:201–206

[CR46] McCune B, Mefford MJ (2011) PC-ORD: multivariate analysis of ecological data. Gleneden Beach, OR, USA: M Software

[CR47] McGonigle TP, Miller MH, Evans DG et al (1990) A new method which gives an objective measure of colonization of roots by vesicular—arbuscular mycorrhizal fungi. New Phytol 115:495–501 33874272 10.1111/j.1469-8137.1990.tb00476.x

[CR48] Merrild MP, Ambus P, Rosendahl S, Jakobsen I (2013) Common arbuscular mycorrhizal networks amplify competition for phosphorus between seedlings and established plants. New Phytol 200:229–240. 10.1111/nph.12351 23738787 10.1111/nph.12351

[CR49] Miller RM, Reinhardt DR, Jastrow JD (1995) External hyphal production of vesicular-arbuscular mycorrhizal fungi in pasture and tallgrass prairie communities. Oecologia 103(1):17–23 28306940 10.1007/BF00328420

[CR50] Montesinos-Navarro A, Verdú M, Querejeta JI et al (2016) Soil fungi promote nitrogen transfer among plants involved in long-lasting facilitative interactions. Perspect Plant Ecol, Evol Syst 18:45–51. 10.1016/j.ppees.2016.01.004

[CR51] Moreno Jiménez E, Ferrol N, Corradi N, et al (2024) The potential of arbuscular mycorrhizal fungi to enhance metallic micronutrient uptake and mitigate food contamination in agriculture: prospects and challenges. New Phytologist. 10.1111/nph.19269 10.1111/nph.19269 37737033

[CR52] Oehl F, de Souza FA, Sieverding E (2008) Revision of Scutellospora and description of five new genera and three new families in the arbuscular mycorrhiza-forming Glomeromycetes. Mycotaxon 106:311–360

[CR53] Ouledali S, Ennajeh M, Zrig A et al (2018) Estimating the contribution of arbuscular mycorrhizal fungi to drought tolerance of potted olive trees (*Olea europaea*). Acta Physiol Plant 40:81. 10.1007/s11738-018-2656-1

[CR54] Pagano MC, Zandavalli RB, Araújo FS (2013) Biodiversity of arbuscular mycorrhizas in three vegetational types from the semiarid of Ceará State, Brazil. Appl Soil Ecol 67:37–46. 10.1016/j.apsoil.2013.02.007

[CR55] Pauwels R, Jansa J, Püschel D et al (2020) Root growth and presence of *Rhizophagus irregularis* distinctly alter substrate hydraulic properties in a model system with *Medicago truncatula*. Plant Soil 457:131–151. 10.1007/s11104-020-04723-w

[CR56] Pauwels R, Graefe J, Bitterlich M (2023) An arbuscular mycorrhizal fungus alters soil water retention and hydraulic conductivity in a soil texture specific way. Mycorrhiza 33:165–179. 10.1007/s00572-023-01106-8 36976365 10.1007/s00572-023-01106-8 PMC10244285

[CR57] Püschel D, Bitterlich M, Rydlová J, Jansa J (2020) Facilitation of plant water uptake by an arbuscular mycorrhizal fungus: a Gordian knot of roots and hyphae. Mycorrhiza 30:299–313 32253570 10.1007/s00572-020-00949-9

[CR58] Püschel D, Bitterlich M, Rydlová J, Jansa J (2021) Drought accentuates the role of mycorrhiza in phosphorus uptake. Soil Biol Biochem 157:108243

[CR59] Püschel D, Bitterlich M, Rydlová J et al (2023) Benefits in plant N uptake via the mycorrhizal pathway in ample soil moisture persist under severe drought. Soil Biol Biochem 187:109220. 10.1016/j.soilbio.2023.109220

[CR60] Puy J, Carmona CP, Hiiesalu I et al (2022) Mycorrhizal symbiosis alleviates plant water deficit within and across generations via phenotypic plasticity. J Ecol 110:262–276. 10.1111/1365-2745.13810

[CR61] Querejeta J, Egerton-Warburton LM, Allen MF (2003) Direct nocturnal water transfer from oaks to their mycorrhizal symbionts during severe soil drying. Oecologia 134:55–64. 10.1007/s00442-002-1078-2 12647179 10.1007/s00442-002-1078-2

[CR62] Quiroga G, Erice G, Ding L et al (2019) The arbuscular mycorrhizal symbiosis regulates aquaporins activity and improves root cell water permeability in maize plants subjected to water stress. Plant, Cell Environ 42:2274–2290. 10.1111/pce.13551 30916398 10.1111/pce.13551

[CR63] Ruiz-Lozano JM (2003) Arbuscular mycorrhizal symbiosis and alleviation of osmotic stress. New Perspect Mol Stud Mycorrhiza 13:309–317 10.1007/s00572-003-0237-6 12690537

[CR64] Ruiz-Lozano JM, Azcón R (1995) Hyphal contribution to water uptake in mycorrhizal plants as affected by the fungal species and water status. Physiol Plant 95:472–478. 10.1111/j.1399-3054.1995.tb00865.x

[CR65] Ruth B, Khalvati M, Schmidhalter U (2011) Quantification of mycorrhizal water uptake via high-resolution on-line water content sensors. Plant Soil 342:459–468. 10.1007/s11104-010-0709-3

[CR66] Saharan K, Schütz L, Kahmen A, et al (2018) Finger millet growth and nutrient uptake is improved in intercropping with pigeon pea through “biofertilization” and “bioirrigation” mediated by arbuscular mycorrhizal fungi and plant growth promoting rhizobacteria. Front Environ Sci 6. 10.3389/fenvs.2018.00046

[CR67] Schüssler A, Walker C (2010) The glomeromycota: a species list with new families and new genera. amf-phylogeny.com. Accessed 22 Feb 2023

[CR68] Selosse M-A, Richard F, He X, Simard SW (2006) Mycorrhizal networks: des liaisons dangereuses? Trends Ecol Evol 21:621–628. 10.1016/j.tree.2006.07.003 16843567 10.1016/j.tree.2006.07.003

[CR69] Singh D, Mathimaran N, Boller T, Kahmen A (2019) Bioirrigation: a common mycorrhizal network facilitates the water transfer from deep-rooted pigeon pea to shallow-rooted finger millet under drought. Plant Soil 440:277–292. 10.1007/s11104-019-04082-1

[CR70] Singh D, Mathimaran N, Boller T, Kahmen A (2020) Deep-rooted pigeon pea promotes the water relations and survival of shallow-rooted finger millet during drought—Despite strong competitive interactions at ambient water availability. PLoS ONE 15:e0228993. 10.1371/journal.pone.0228993 32053664 10.1371/journal.pone.0228993 PMC7018066

[CR71] Smith FA, Jakobsen I, Smith SE (2000) Spatial differences in acquisition of soil phosphate between two arbuscular mycorrhizal fungi in symbiosis with *Medicago truncatula*. New Phytol 147:357–366

[CR72] Smith SE, Read DJ (2010) Mycorrhizal symbiosis. Academic Press, London, UK

[CR73] Symanczik S, Lehmann MF, Wiemken A et al (2018) Effects of two contrasted arbuscular mycorrhizal fungal isolates on nutrient uptake by *Sorghum bicolor* under drought. Mycorrhiza 28:779–785. 10.1007/s00572-018-0853-9 30006910 10.1007/s00572-018-0853-9

[CR74] Taiz L, Zeiger E, Møller IM, Murphy A (2015) Plant physiology and development. Sinauer Associates Inc., Sunderland, MA, U.S.A.

[CR75] Taylor JR (1982) An introduction to error analysis. Edited by Eugene D. Commins. Sausalito. CA USA: University Science Books

[CR76] Tereucán G, Ruiz A, Nahuelcura J et al (2022) Shifts in biochemical and physiological responses by the inoculation of arbuscular mycorrhizal fungi in *Triticum aestivum* growing under drought conditions. J Sci Food Agric 102:1927–1938. 10.1002/jsfa.11530 34510460 10.1002/jsfa.11530

[CR77] Thonar C, Schnepf A, Frossard E et al (2011) Traits related to differences in function among three arbuscular mycorrhizal fungi. Plant Soil 339:231–245

[CR78] Uehlein N, Fileschi K, Eckert M et al (2007) Arbuscular mycorrhizal symbiosis and plant aquaporin expression. Phytochemistry 68:122–129. 10.1016/j.phytochem.2006.09.033 17109903 10.1016/j.phytochem.2006.09.033

[CR79] van der Heijden MG, Martin FM, Selosse M-A, Sanders IR (2015) Mycorrhizal ecology and evolution: the past, the present, and the future. New Phytol 205:1406–1423 25639293 10.1111/nph.13288

[CR80] von Liebig J (1840) Die organische Chemie in ihrer Anwendung auf Agricultur und Physiologie. Vieweg

[CR81] Walder F, van der Heijden MG (2015) Regulation of resource exchange in the arbuscular mycorrhizal symbiosis. Nat Plants 1:1–7 10.1038/nplants.2015.159 27251530

[CR82] Walder F, Niemann H, Natarajan M et al (2012) Mycorrhizal Networks: Common Goods of Plants Shared under Unequal Terms of Trade. Plant Physiol 159:789–797. 10.1104/pp.112.195727 22517410 10.1104/pp.112.195727 PMC3375941

[CR83] Walder F, Boller T, Wiemken A, Courty P-E (2016) Regulation of plants’ phosphate uptake in common mycorrhizal networks: Role of intraradical fungal phosphate transporters. Plant Signal Behav 11:e1131372. 10.1080/15592324.2015.1131372 26751110 10.1080/15592324.2015.1131372 PMC4883902

[CR84] Walker C, Trappe J (1981) *Acaulospora spinosa* sp. nov. with a key to the species of Acaulospora. Mycotaxon 12:515–521

[CR85] Weremijewicz J, Janos DP (2013) Common mycorrhizal networks amplify size inequality in *Andropogon gerardii* monocultures. New Phytol 198:203–213 23356215 10.1111/nph.12125

[CR86] Weremijewicz J, Janos DP (2019) Investigation of plant interactions across common mycorrhizal networks using rotated cores. JoVE (Journal of Visualized Experiments) (145):e59338 10.3791/59338 30985758

[CR87] Weremijewicz J, Seto K (2016) Mycorrhizas influence functional traits of two tallgrass prairie species. Ecol Evol 6:3977–3990. 10.1002/ece3.2129 27516857 10.1002/ece3.2129 PMC4874859

[CR88] Weremijewicz J, da Silveira LO, Janos DP (2018) Arbuscular common mycorrhizal networks mediate intra-and interspecific interactions of two prairie grasses. Mycorrhiza 28:71–83 28986642 10.1007/s00572-017-0801-0

[CR89] Weremijewicz J, Sternberg L da SLO, Janos DP (2016) Common mycorrhizal networks amplify competition by preferential mineral nutrient allocation to large host plants. New Phytologist 212:461–471 10.1111/nph.14041 27265515

[CR90] Wesseling J, Van Wijk WR, Fireman M et al (1957) Land drainage in relation to soils and crops. Drain Agric Lands 7:461–578

[CR91] Wipf D, Krajinski F, van Tuinen D et al (2019) Trading on the arbuscular mycorrhiza market: from arbuscules to common mycorrhizal networks. New Phytol 223:1127–1142 30843207 10.1111/nph.15775

[CR92] Wu H-H, Zou Y-N, Rahman MM et al (2017) Mycorrhizas alter sucrose and proline metabolism in trifoliate orange exposed to drought stress. Sci Rep 7:1–10 28181575 10.1038/srep42389 PMC5299426

[CR93] Wu C, Bi Y, Zhu W (2024) Is the amount of water transported by arbuscular mycorrhizal fungal hyphae negligible? Insights from a compartmentalized experimental study. Plant Soil 499:537–552. 10.1007/s11104-024-06477-1

[CR94] Yang Y, Tang M, Sulpice R et al (2014) Arbuscular Mycorrhizal Fungi Alter Fractal Dimension Characteristics of *Robinia pseudoacacia* L. Seedlings Through Regulating Plant Growth, Leaf Water Status, Photosynthesis, and Nutrient Concentration Under Drought Stress. J Plant Growth Regul 33:612–625. 10.1007/s00344-013-9410-0

[CR95] Zheng C, Ji B, Zhang J et al (2015) Shading decreases plant carbon preferential allocation towards the most beneficial mycorrhizal mutualist. New Phytol 205:361–368. 10.1111/nph.13025 25243653 10.1111/nph.13025

[CR96] Zou Y-N, Wu Q-S, Kuča K (2021) Unravelling the role of arbuscular mycorrhizal fungi in mitigating the oxidative burst of plants under drought stress. Plant Biol 23:50–57. 10.1111/plb.13161 32745347 10.1111/plb.13161

