# Supplementary material for: Common mycorrhizal networks improve survival and mediate facilitative plant interactions among Andropogon gerardii seedlings under drought stress
Source: Mycorrhiza. 2025 Feb 3;35(1):8. doi: 10.1007/s00572-025-01181-z (PMC11790713; doi:10.1007/s00572-025-01181-z)
Supplement: Supplementary file 1 — Supplementary file1 (DOCX 787 KB) [file 572_2025_1181_MOESM1_ESM.docx]

**Supplementary Information (SI)**

**SI Table 1**. Mineral nutrient concentrations and properties of the crushed glass mixture in the interstices between cone-tainers and soil within cone-tainers of each pot.

| Soil characteristic* | Interstitial crushed glass mixture | Soil (within cone-tainers) |  |
| --- | --- | --- | --- |
| Phosphorus (μg g^-1^) | 6.0 (Bray) | 10.2 (Olsen) |  |
| Potassium (μg g^-1^) | 24.0 | 38 |  |
| Nitrate (μg g^-1^) | 0.7 | 9.4 |  |
| Ammonium (μg g^-1^) | 0.9 | 5.35 |  |
| Magnesium (μg g^-1^) | 39.0 | 147.9 |  |
| Calcium (μg g^-1^) | 323.0 | 2511.6 |  |
| Sodium (μg g^-1^) | 90.0 | 54.7 |  |
| Manganese (μg g^-1^) | 1.0 | 1.5 |  |
| Iron (μg g^-1^) | 5.0 | 153.8 |  |
| pH | 7.9 | 7.6 |  |
| Cation Exchange Capacity  (meq 100g^-1^) | 1.0 | 14.22 |  |

**SI Table 2.** Samples size (N; total number of pots), mean shoot dry weight (DW), standard error (SE), coefficient of variation (C.V.), and the range of average plant sizes (Minimum and Maximum) per pot within the Watered, Intact CMNs treatment before and after omission of an outlier that affected normality of the residuals, which violated the two-factor ANOVA

|  | N | Mean Shoot DW per pot | SE | C.V. | Minimum | Maximum |
| --- | --- | --- | --- | --- | --- | --- |
| Before outlier omission | 10 | 0.0287 | 0.0033 | 35.75 | 0.0175 | 0.055 |
| After outlier omission | 9 | 0.0258 | 0.0016 | 18.35 | 0.0175 | 0.034 |

**SI Table 3.** Statistical analysis results from tests for assumptions (Levene’s test and Shapiro-Wilk test) of a two-factor ANOVA, as well as Two-Factor ANOVA results before and after an outlier was removed from the Watered, Intact CMNs treatment

|  | Test for Homogenous variance | | Test for Normal Residuals | | Two-Factor ANVOA | | | |
| --- | --- | --- | --- | --- | --- | --- | --- | --- |
| Data Management | Levene’s Test *F* | *P* | Shapiro-Wilk *W* | *P* | Factor | *df* | *F* | *P* |
| Before outlier omission | 1.10 | 0.3642 | 0.8734 | 0.0006 | CMNs | 1,33 | 6.84 | 0.0133 |
|  |  |  |  |  | Water |  | 42.73 | <0.0001 |
|  |  |  |  |  | CMNs x Water |  | 0.46 | 0.5001 |
| After outlier omission | 2.23 | 0.1036 | 0.9813 | 0.7899 | CMNs | 1,32 | 7.23 | 0.0113 |
|  |  |  |  |  | Water |  | 64.51 | <0.0001 |
|  |  |  |  |  | CMNs x Water |  | 3.26 | 0.0806 |

**SI Table 4.** Principle components analysis (PCA) percentage of variance represented by the first axis, loadings on Axis 1 for the four PCA-summarized neighbor categories, and the correlation between Axis 1 and target whole-plant dry weights prior to rotation of Axis 1 for treatments with drought or watered conditions and with severed or intact common mycorrhizal networks (CMNs). For all treatments, the first axis was the only significant axis in explaining the variance among neighboring plants

|  | Proportion of variance (*P**) | Pearson's correlation coefficient with Axis 1†, *r* | | | | |
| --- | --- | --- | --- | --- | --- | --- |
| Treatment | Axis 1 | Largest neighbor | 2nd largest neighbor | 3rd largest neighbor | 4^th^ largest neighbor | Targets |
| Intact CMNs, Watered | 80.5% | -0.921 | -0.946 | -0.904 | -0.744 | -0.517 |
|  | (0.001) |  |  |  |  |  |
| Intact CMNs, Drought | 92.9% | -0.956 | -0.983 | -0.986 | -0.897 | -0.561 |
|  | (0.001) |  |  |  |  |  |
| Severed CMNs, Watered | 68.0% | 0.802 | 0.108 | -0.975 | 0.953 | 0.007 |
|  | (0.011) |  |  |  |  |  |
| Severed CMNs, Drought | 76.6% | -0.966 | -0.971 | -0.5 | -0.529 | -0.101 |
|  | (0.005) |  |  |  |  |  |

**SI Table 5.** Two-way ANOVA results (degrees of freedom, *F*-statistics, and associated *P* values) for the main effect of watering conditions, CMN treatments (intact or severed), and their interaction on shoot mineral nutrient concentrations of pooled samples based upon shoot dry weights of *Andropogon gerardii* seedlings

|  | Treatment | | Value | |  | Main Effect Water | | Main Effect CMNs | | Water x CMNs | |
| --- | --- | --- | --- | --- | --- | --- | --- | --- | --- | --- | --- |
| Nutrient (unit) | Watering Condition | CMNs | Mean | SD | *df* | *F* | *P* | *F* | *P* | *F* | *P* |
| P (%) | Watered | Intact | 0.190 | 0.041 | 1,33 | 0.00 | 0.967 | 3.12 | 0.086 | 1.50 | 0.230 |
|  |  | Severed | 0.165 | 0.022 |  |  |  |  |  |  |  |
|  | Drought | Intact | 0.176 | 0.043 |  |  |  |  |  |  |  |
|  |  | Severed | 0.146 | 0.033 |  |  |  |  |  |  |  |
| K (%) | Watered | Intact | 0.960 | 0.233 | 1,33 | 0.78 | 0.384 | 2.09 | 0.158 | 0.04 | 0.853 |
|  |  | Severed | 1.140 | 0.187 |  |  |  |  |  |  |  |
|  | Drought | Intact | 1.014 | 0.161 |  |  |  |  |  |  |  |
|  |  | Severed | 1.057 | 0.164 |  |  |  |  |  |  |  |
| Ca (%) | Watered | Intact | 0.882 | 0.215 | 1,33 | **5.13** | **0.030** | 2.57 | 0.118 | 2.74 | 0.108 |
|  |  | Severed | 1.170 | 0.277 |  |  |  |  |  |  |  |
|  | Drought | Intact | 0.928 | 0.173 |  |  |  |  |  |  |  |
|  |  | Severed | 0.878 | 0.134 |  |  |  |  |  |  |  |
| Mg (%) | Watered | Intact | 0.323 | 0.046 | 1,33 | 0.04 | 0.835 | 0.05 | 0.819 | 0.00 | 0.976 |
|  |  | Severed | 0.323 | 0.042 |  |  |  |  |  |  |  |
|  | Drought | Intact | 0.326 | 0.038 |  |  |  |  |  |  |  |
|  |  | Severed | 0.319 | 0.040 |  |  |  |  |  |  |  |
| S* (%) | Watered | Intact | 0.171^AB^ | 0.036 | 1,32 | **15.66** | **0.004** | 2.19 | 0.149 | **5.47** | **0.026** |
|  |  | Severed | 0.251^A^ | 0.023 |  |  |  |  |  |  |  |
|  | Drought | Intact | 0.191^B^ | 0.026 |  |  |  |  |  |  |  |
|  |  | Severed | 0.159^C^ | 0.020 |  |  |  |  |  |  |  |
| Cu* (ppm) | Watered | Intact | 21.2 | 4.12 | 1,31 | 2.10 | 0.157 | 2.40 | 0.132 | 0.00 | 0.960 |
|  |  | Severed | 25.9 | 2.97 |  |  |  |  |  |  |  |
|  | Drought | Intact | 23.3 | 3.58 |  |  |  |  |  |  |  |
|  |  | Severed | 23.4 | 2.66 |  |  |  |  |  |  |  |
| Fe* (ppm) | Watered | Intact | 913^C^ | 417 | 1,33 | **24.59** | **<0.0001** | **37.18** | **<0.0001** | **12.71** | **0.001** |
|  |  | Severed | 5532^A^ | 2505 |  |  |  |  |  |  |  |
|  | Drought | Intact | 1134^BC^ | 472 |  |  |  |  |  |  |  |
|  |  | Severed | 1420^B^ | 718 |  |  |  |  |  |  |  |
| Mn* (ppm) | Watered | Intact | 20.5 | 9.4 | 1,33 | **40.84** | **<0.0001** | 0.32 | 0.574 | 3.17 | 0.084 |
|  |  | Severed | 63.4 | 15.8 |  |  |  |  |  |  |  |
|  | Drought | Intact | 54.0 | 27.7 |  |  |  |  |  |  |  |
|  |  | Severed | 14.0 | 11.9 |  |  |  |  |  |  |  |
| Zn* (ppm) | Watered | Intact | 56.3^B^ | 16.6 | 1,32 | 1.83 | 0.186 | **4.36** | **0.045** | **6.49** | **0.016** |
|  |  | Severed | 48.1^B^ | 3.8 |  |  |  |  |  |  |  |
|  | Drought | Intact | 119.6^A^ | 82.2 |  |  |  |  |  |  |  |
|  |  | Severed | 61.8^B^ | 22.7 |  |  |  |  |  |  |  |

* indicates data were transformed (as described in Statistical Methods) prior to analysis

**SI Table 6.** Two-way Repeated Measures ANOVA results (degrees of freedom, F-statistics, and associated P values) for the main effects of CMNs (intact or severed CMNs) and watering treatments (watered or drought) and their interaction on the rate of drying (change in % substrate moisture per day) of cone-tainer soil and interstitial substrate soil moisture over time

| Substrate | Factor | Df | F | P |
| --- | --- | --- | --- | --- |
| Cone-tainer soil | CMN | 1, 33 | 0.17 | 0.6869 |
|  | Water | 1, 33 | 2.90 | 0.0978 |
|  | CMN x Water | 1, 33 | 2.20 | 0.1477 |
|  | Time | 17, 561 | 107.16 | < 0.00001* |
|  | CMN x Time | 17, 561 | 2.76 | 0.0104* |
|  | Water x Time | 17, 561 | 28.49 | < 0.00001* |
|  | CMN x Water x Time | 17, 561 | 3.77 | 0.0009* |
| Interstitial Substrate | CMN | 1, 33 | 0.75 | 0.3936 |
|  | Water | 1, 33 | 6.73 | 0.0140 |
|  | CMN x Water | 1, 33 | 0.54 | 0.4690 |
|  | Time | 17, 561 | 253.01 | < 0.00001* |
|  | CMN x Time | 17, 561 | 1.81 | 0.0920* |
|  | Water x Time | 17, 561 | 19.68 | < 0.00001* |
|  | CMN x Water x Time | 17, 561 | 1.13 | 0.3481* |

* indicates a Greenhouse-Geisser Corrected P-value for non-sphericity after a significant Mauchly’s Sphericity Assumption test (P<0.05).

**
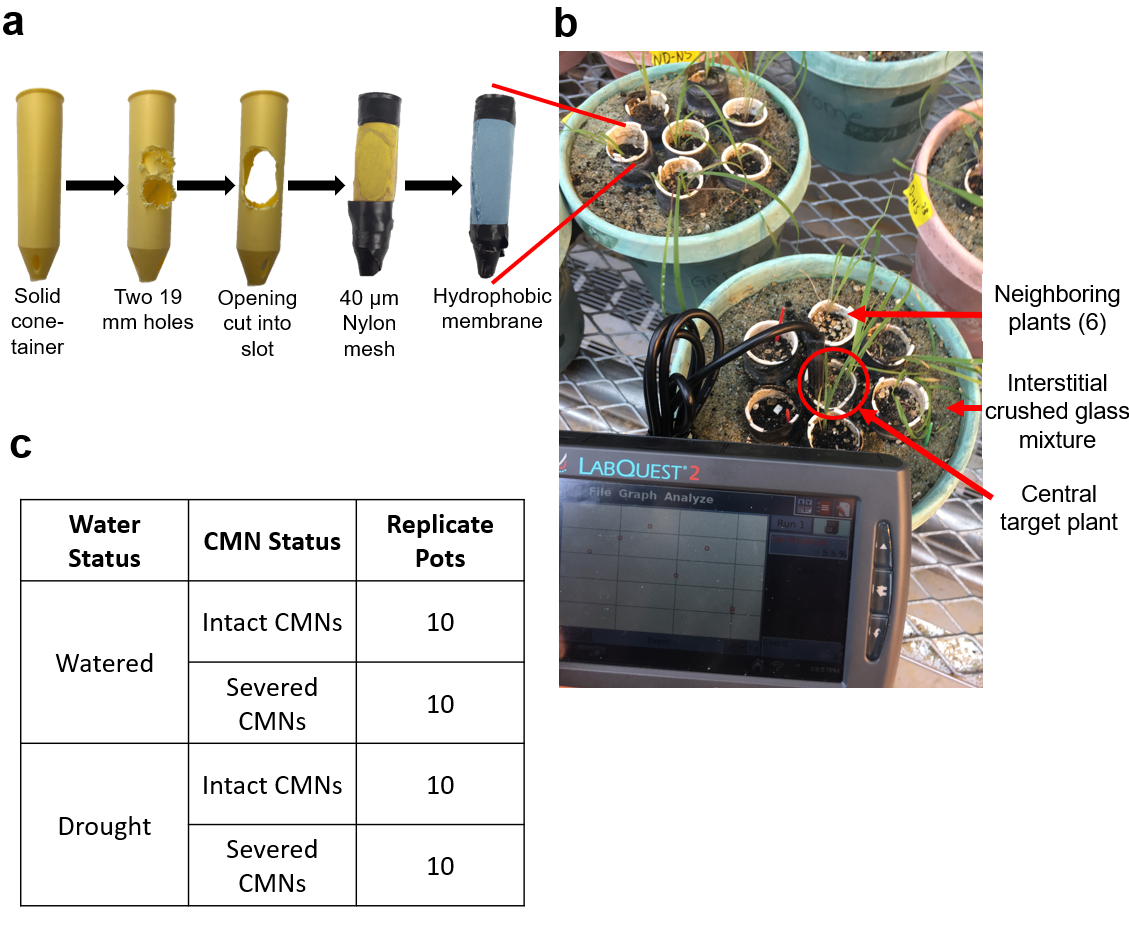
SI Figure 1**: Experiment design of modified Ray Leach Cone-tainers ™ (**a**) as they were modified with openings, 40 µm nylon silk screen mesh, and a Gore-Tex ™ hydrophobic membrane. Individual *Andropogon gerardii* seedlings were grown in modified cone-tainers with nylon mesh and hydrophobic membranes that were placed into pots (**b**) in which a central target plant was surrounded by six equidistant neighboring. The interstices were filled with a 4:1 nutrient-poor fine crushed glass (20-40 grade, Harsco Corporation, Camp Hill, PA, USA) and glass bead (12-20 grade, Industrial Supply, Inc, Twin Falls, ID, USA) mixture. Modified cone-tainers were filled with a sandy soil mixture. Nutrient concentrations of media can be found in SI Table 1. The photo (**b**) additionally depicts the frequent soil moisture measurements that were conducted with using a LabQuest 2 Vernier Data Logger and soil moisture probe (Beaverton, OR, USA) on both the interstitial crushed glass mixture and soil within cone-tainers. If the values fell below our target range, water was added to the pots by hand. (**c**) The experiment was a factorial experiment in which watering was manipulated to be under well-watered (25-30% soil moisture) or drought (15-20% soil moisture) conditions and CMNs were kept either intact or severed through weekly rotation of cone-tainers.
